# Bis(benzene-1,2-diamine-κ^2^ *N*,*N*′)(sulfato-κ*O*)copper(II) monohydrate

**DOI:** 10.1107/S1600536812041967

**Published:** 2012-10-27

**Authors:** Yacine Djebli, Sihem Boufas, Leila Bencharif, Thierry Roisnel, Mustafa Bencharif

**Affiliations:** a Université Mentouri de Constantine, 25000 Constantine, Algeria; b Université 20 Aout 1955, 21000 Skikda, Algeria; c Sciences Chimiques de Rennes (UMR CNRS 6226), Université de Rennes 1, Avenue du Général Leclerc, 35042 Rennes Cedex, France

## Abstract

The title complex, [Cu(SO_4_)(C_6_H_8_N_2_)_2_]·H_2_O, was obtained under hydro­thermal conditions. The Cu^II^ ion is five-coordinated in a distorted square-pyramidal manner by four N atoms from two benzene-1,2-diamine ligands at the base and one O atom from a monodentate sulfate anion at the apex of the coordination polyhedron. N—H⋯O hydrogen bonding between the amino functions and the sulfate groups leads to the formation of layers parallel to (001). C—H⋯O hydrogen bonding inter­actions between the layers consolidate the three-dimensional set-up. There are voids in the structure filled with lattice water mol­ecules that are disordered over three sites in a 0.430 (6):0.270 (6):0.300 (6) ratio.

## Related literature

For bio-inorganic chemistry and the coordination chemistry of copper(II), see: Datta *et al.* (2008 ▶); Diallo *et al.* (2008 ▶); Khalaji *et al.* (2009 ▶). For graph-set notation, see: Bernstein *et al.* (1995 ▶); Etter *et al.* (1990 ▶).
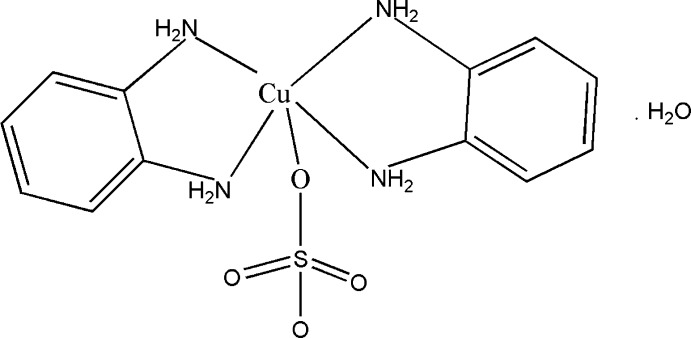


## Experimental

### 

#### Crystal data

[Cu(SO_4_)(C_6_H_8_N_2_)_2_]·H_2_O
*M* *_r_* = 393.90
Orthorhombic, 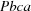

*a* = 18.6794 (4) Å
*b* = 7.5317 (2) Å
*c* = 21.9757 (5) Å
*V* = 3091.71 (13) Å^3^
*Z* = 8
Mo *K*α radiation
μ = 1.58 mm^−1^
*T* = 120 K
0.38 × 0.15 × 0.05 mm

#### Data collection

Nonius KappaCCD diffractometer
Absorption correction: multi-scan (*SORTAV*; Blessing, 1995 ▶) *T*_min_ = 0.543, *T*_max_ = 0.924
42067 measured reflections
3555 independent reflections
2957 reflections with *I* > 2σ(*I*)
*R*_int_ = 0.057

#### Refinement

*R*[*F* ^2^ > 2σ(*F* ^2^)] = 0.034
*wR*(*F* ^2^) = 0.097
*S* = 1.08
3555 reflections
212 parameters
1 restraint
H-atom parameters constrained
Δρ_max_ = 1.29 e Å^−3^
Δρ_min_ = −0.64 e Å^−3^

### 

Data collection: *COLLECT* (Nonius, 2002 ▶); cell refinement: *DENZO-SMN* (Otwinowski & Minor, 1997 ▶); data reduction: *EVALCCD* (Duisenberg *et al.*, 2003 ▶); program(s) used to solve structure: *SIR97* (Altomare *et al.*, 1999 ▶); program(s) used to refine structure: *SHELXL97* (Sheldrick, 2008 ▶); molecular graphics: *ORTEPIII* (Farrugia, 1997 ▶); software used to prepare material for publication: *WinGX* (Farrugia, 1999 ▶) and *PARST* (Nardelli, 1995 ▶).

## Supplementary Material

Crystal structure: contains datablock(s) I, global. DOI: 10.1107/S1600536812041967/wm2682sup1.cif

Structure factors: contains datablock(s) I. DOI: 10.1107/S1600536812041967/wm2682Isup2.hkl

Additional supplementary materials:  crystallographic information; 3D view; checkCIF report

## Figures and Tables

**Table 1:** Selected bond lengths (Å)

Cu1—N2	2.014 (2)
Cu1—N4	2.020 (2)
Cu1—N1	2.022 (2)
Cu1—N3	2.023 (2)
Cu1—O1	2.2433 (18)

**Table 2:** Hydrogen-bond geometry (Å, °)

*D*—H⋯*A*	*D*—H	H⋯*A*	*D*⋯*A*	*D*—H⋯*A*
N1—H1*A*⋯O4^i^	0.90	2.03	2.904 (3)	164
N1—H1*B*⋯O2	0.90	2.06	2.873 (4)	149
N2—H2*A*⋯O3^ii^	0.90	2.16	3.059 (4)	174
N2—H2*B*⋯O3^iii^	0.90	2.02	2.890 (4)	161
N3—H3*A*⋯O3^iii^	0.90	2.45	3.188 (4)	139
N3—H3*A*⋯O4^iii^	0.90	2.24	3.068 (4)	153
N4—H4*A*⋯O4	0.90	2.37	3.202 (4)	153
N4—H4*B*⋯O2^i^	0.90	1.96	2.825 (4)	160
C4—H4⋯O4^iv^	0.93	2.59	3.512 (4)	172

Symmetry codes: (i) 
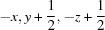
; (ii) 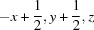; (iii) 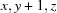; (iv) 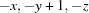.

**Fig. 1.**
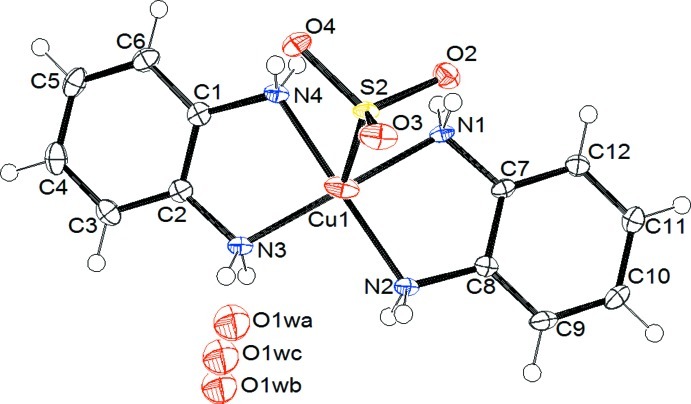
The molecular structure of the title compound drawn with displacement parameters at the 50% probability level.

**Fig. 2.**
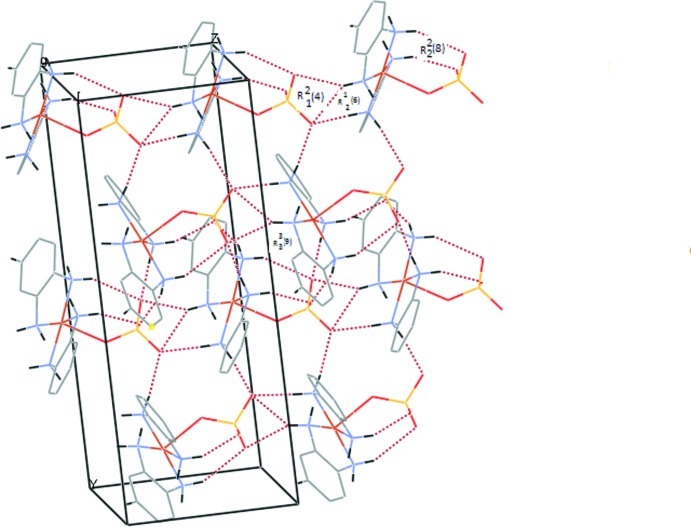
Part of the crystal structure of (I), showing the formation of R22(8), R21(4),hy2596 R12(6) and R33(9) rings. Hydrogen bonds are shown as dashed lines. Hydrogen atoms not involved in the motif have been omitted for clarity.

## supplementary crystallographic information

### Comment

Copper(II) complexes have been widely investigated in both bioinorganic chemistry and coordination chemistry (Datta *et al.*, 2008; Diallo *et al.*, 2008; Khalaji *et al.*, 2009) due to the important role of copper in living organisms and its peculiar coordination chemistry, i.e. the Jahn-Teller distortion. We report here the structure of the title mononuclear copper(II) complex, [Cu(SO_4_)(C_6_H_8_N_2_)_2_]^.^H_2_O.

As shown in Fig. 1, the complex molecule exhibits copper(II) in a distorted [4 + 1] square-pyramidal coordination environment. The basal plane is defined by the amino nitrogen donors of the benzene-1,2-diamine ligand, while the apical position is occupied by one oxygen atom of the SO_4_^2-^ anion. As expected, the organic moieties are approximately planar. In the organic moiety bonded to the copper atom with N1 and N2, the r.m.s. deviation for the non-H atoms is 0.0103 Å, with the maximum deviation of N1 from the mean plane being -0.018 (2) Å. The 1,2 benzene-1,2-diamine molecule linked to Cu(II) with N3 and N4 has a r.m.s deviation for non-H atoms of 0.0173 Å with a maximum deviation from the mean plane being -0.027 (2) Å (for N4). In the crystal structure, π···π stacking interactions occur between adjacent rings, with centroid-centroid separations of 3.583 (2) Å for *Cg*1···*Cg*2^i^ (*Cg*1 and *Cg*2 are the centroids of the C1—C6 and C7—C12 rings, respectively; symmetry code: (i) (-*x*, -*y*, -*z*)).

In the title compound, the individual molecules are linked by N—H···O hydrogen bonds into a two-dimensional network parallel to (001). In the layers alternating *R*^2^_2_(8), *R*^2^_1_(4), *R*^1^_2_(6) and dual *R*^3^_3_(9) (Bernstein *et al.*, 1995) hydrogen-bonded motifs are formed. These layers are linked by means of C15—H15···O22 hydrogen bonds. In terms of graph-set analysis (Etter *et al.*, 1990; Bernstein *et al.*, 1995), the descriptor for this pattern is *R*^2^_4_(16).

In the structure voids with an approximate volume of 62 Å^3^ are present. These voids are filled with a water molecule disordered over three positions with an occupancy ratio of 0.4320 (6):0.270 (6):0.300 (6). Although the H positions of the disordered water molecule could not be located, O···O and O···N interactions suggest likewise an involvement in hydrogen bonding, e.g. N3···O1WC = 2.98 (4) Å, O1···O1WA = 2.73 (3) Å.

### Experimental

Benzene-1,2-diamine (0.2 mmol) and CuSO_4_.5H_2_O (0.1 mmol) were dissolved in H_2_O (5 ml). The mixture was stirred for 16 h at room temperature to give a violet solution. After allowing the resulting solution to stand in air, violet plate-shaped crystals of the compound were formed on slow evaporation of the solvent.

### Refinement

C—H and N—H hydrogen atoms were placed in calculated positions and refined as riding atoms with C—H distances of 0.95 Å with *U*_iso_(H) = 1.2Ueq(C) and N—H distances of 0.92 Å, with *U*_iso_(H) = 1.2Ueq(N).

The water molecule was localized in a difference map and was interpreted as disordered over three positions with the site-occupancy ratio of 0.220 (6):0.270 (6):0.300 (6). The H atoms on the disordered water molecules were not included in the refinement.

### Crystal data

**Table:**

[Cu(SO_4_)(C_6_H_8_N_2_)_2_]·H_2_O	*F*(000) = 1624
*M**_r_* = 393.90	*D*_x_ = 1.693 Mg m^−^^3^
Orthorhombic, *P**b**c**a*	Mo *K*α radiation, λ = 0.71073 Å
Hall symbol: -P 2ac 2ab	Cell parameters from 102968 reflections
*a* = 18.6794 (4) Å	θ = 2.9–27.5°
*b* = 7.5317 (2) Å	µ = 1.58 mm^−^^1^
*c* = 21.9757 (5) Å	*T* = 120 K
*V* = 3091.71 (13) Å^3^	Plate, violet
*Z* = 8	0.38 × 0.15 × 0.05 mm

### Data collection

**Table:**

Nonius KappaCCD diffractometer	3555 independent reflections
Radiation source: Enraf Nonius FR590	2957 reflections with *I* > 2σ(*I*)
Graphite monochromator	*R*_int_ = 0.057
Detector resolution: 9 pixels mm^-1^	θ_max_ = 27.5°, θ_min_ = 3.1°
CCD rotation images, thin slices scans	*h* = −24→24
Absorption correction: multi-scan (*SORTAV*; Blessing, 1995)	*k* = −9→9
*T*_min_ = 0.543, *T*_max_ = 0.924	*l* = −28→28
42067 measured reflections	

### Refinement

**Table:**

Refinement on *F*^2^	Primary atom site location: structure-invariant direct methods
Least-squares matrix: full	Secondary atom site location: difference Fourier map
*R*[*F*^2^ > 2σ(*F*^2^)] = 0.034	Hydrogen site location: inferred from neighbouring sites
*wR*(*F*^2^) = 0.097	H-atom parameters constrained
*S* = 1.08	*w* = 1/[σ^2^(*F*_o_^2^) + (0.0455*P*)^2^ + 5.6842*P*] where *P* = (*F*_o_^2^ + 2*F*_c_^2^)/3
3555 reflections	(Δ/σ)_max_ = 0.005
212 parameters	Δρ_max_ = 1.29 e Å^−^^3^
1 restraint	Δρ_min_ = −0.64 e Å^−^^3^

### Special details

**Table:**

Geometry. All e.s.d.'s (except the e.s.d. in the dihedral angle between two l.s. planes) are estimated using the full covariance matrix. The cell e.s.d.'s are taken into account individually in the estimation of e.s.d.'s in distances, angles and torsion angles; correlations between e.s.d.'s in cell parameters are only used when they are defined by crystal symmetry. An approximate (isotropic) treatment of cell e.s.d.'s is used for estimating e.s.d.'s involving l.s. planes.

### Fractional atomic coordinates and isotropic or equivalent isotropic displacement parameters (Å^2^)

**Table:**

	*x*	*y*	*z*	*U*_iso_*/*U*_eq_	Occ. (<1)
Cu1	0.088690 (15)	0.58928 (4)	0.199850 (13)	0.01191 (11)	
S2	0.12634 (3)	0.15921 (8)	0.18987 (3)	0.01166 (14)	
O4	0.06308 (9)	0.1072 (3)	0.15332 (8)	0.0193 (4)	
O1	0.14861 (9)	0.3423 (2)	0.17409 (8)	0.0174 (4)	
O2	0.10796 (10)	0.1495 (3)	0.25522 (8)	0.0192 (4)	
O3	0.18635 (9)	0.0364 (2)	0.17653 (9)	0.0186 (4)	
N3	0.09519 (11)	0.7199 (3)	0.11963 (10)	0.0155 (4)	
H3A	0.1012	0.8368	0.1265	0.019*	
H3B	0.1332	0.6804	0.0985	0.019*	
N4	−0.00490 (11)	0.4952 (3)	0.16695 (9)	0.0145 (4)	
H4A	−0.0018	0.3769	0.1618	0.017*	
H4B	−0.0403	0.5171	0.1937	0.017*	
N1	0.06897 (11)	0.5075 (3)	0.28588 (9)	0.0135 (4)	
H1A	0.0255	0.5456	0.2977	0.016*	
H1B	0.0692	0.3881	0.2874	0.016*	
N2	0.17909 (11)	0.6930 (3)	0.23489 (9)	0.0141 (4)	
H2A	0.2174	0.6385	0.2185	0.017*	
H2B	0.1817	0.8093	0.2257	0.017*	
C7	0.12327 (12)	0.5774 (3)	0.32658 (11)	0.0134 (5)	
C8	0.17991 (13)	0.6705 (3)	0.30072 (11)	0.0134 (5)	
C3	0.01846 (14)	0.7716 (4)	0.02813 (12)	0.0194 (5)	
H3	0.0529	0.8467	0.0116	0.023*	
C11	0.17574 (14)	0.6137 (4)	0.42584 (12)	0.0189 (5)	
H11	0.1747	0.5948	0.4676	0.023*	
C9	0.23454 (13)	0.7353 (4)	0.33737 (12)	0.0188 (5)	
H9	0.2725	0.7972	0.3201	0.023*	
C1	−0.02133 (13)	0.5799 (3)	0.10905 (11)	0.0149 (5)	
C2	0.03023 (13)	0.6913 (3)	0.08463 (11)	0.0152 (5)	
C10	0.23241 (13)	0.7075 (4)	0.39984 (12)	0.0193 (5)	
H10	0.2689	0.7515	0.4244	0.023*	
C6	−0.08541 (14)	0.5499 (4)	0.07848 (12)	0.0197 (6)	
H6	−0.1204	0.4778	0.0957	0.024*	
C4	−0.04457 (15)	0.7389 (4)	−0.00323 (12)	0.0228 (6)	
H4	−0.0521	0.7903	−0.0412	0.027*	
C12	0.12084 (14)	0.5486 (4)	0.38939 (11)	0.0170 (5)	
H12	0.0829	0.4865	0.4066	0.02*	
C5	−0.09680 (14)	0.6283 (4)	0.02224 (13)	0.0222 (6)	
H5	−0.1393	0.6074	0.0014	0.027*	
O1WA	0.2247 (3)	0.4801 (9)	0.0775 (2)	0.0328 (9)*	0.430 (6)
O1WB	0.2410 (5)	0.6775 (15)	0.0687 (4)	0.0328 (9)*	0.270 (6)
O1WC	0.2338 (4)	0.5778 (17)	0.0693 (4)	0.0328 (9)*	0.300 (6)

### Atomic displacement parameters (Å^2^)

**Table:**

	*U*^11^	*U*^22^	*U*^33^	*U*^12^	*U*^13^	*U*^23^
Cu1	0.00820 (16)	0.01169 (17)	0.01585 (16)	−0.00014 (12)	0.00131 (10)	0.00052 (11)
S2	0.0078 (3)	0.0102 (3)	0.0170 (3)	0.0003 (2)	0.0020 (2)	0.0010 (2)
O4	0.0113 (8)	0.0240 (10)	0.0228 (9)	−0.0026 (8)	−0.0024 (7)	0.0009 (8)
O1	0.0139 (8)	0.0089 (9)	0.0295 (10)	0.0013 (7)	0.0074 (7)	0.0019 (7)
O2	0.0205 (9)	0.0205 (10)	0.0168 (9)	−0.0019 (8)	0.0026 (7)	0.0015 (7)
O3	0.0125 (8)	0.0124 (9)	0.0310 (10)	0.0038 (7)	0.0045 (7)	0.0017 (8)
N3	0.0118 (10)	0.0151 (11)	0.0197 (10)	−0.0023 (8)	0.0023 (8)	0.0007 (9)
N4	0.0114 (9)	0.0130 (10)	0.0190 (10)	−0.0008 (8)	0.0025 (8)	0.0012 (8)
N1	0.0086 (9)	0.0133 (11)	0.0186 (10)	−0.0013 (9)	0.0013 (8)	−0.0021 (8)
N2	0.0104 (9)	0.0119 (10)	0.0201 (10)	−0.0004 (8)	0.0019 (8)	0.0027 (8)
C7	0.0089 (11)	0.0118 (12)	0.0195 (12)	0.0014 (10)	−0.0001 (9)	−0.0015 (9)
C8	0.0115 (11)	0.0106 (12)	0.0182 (12)	0.0032 (10)	0.0015 (9)	0.0009 (9)
C3	0.0212 (13)	0.0186 (13)	0.0183 (12)	0.0041 (11)	0.0046 (10)	0.0007 (10)
C11	0.0182 (12)	0.0205 (14)	0.0180 (12)	0.0031 (11)	−0.0023 (10)	−0.0017 (10)
C9	0.0119 (11)	0.0155 (13)	0.0292 (13)	−0.0022 (11)	−0.0005 (10)	0.0025 (11)
C1	0.0149 (11)	0.0119 (12)	0.0178 (11)	0.0036 (10)	0.0013 (9)	−0.0032 (10)
C2	0.0135 (11)	0.0152 (13)	0.0168 (11)	0.0030 (10)	0.0030 (9)	−0.0038 (10)
C10	0.0139 (12)	0.0181 (13)	0.0260 (13)	0.0015 (10)	−0.0064 (10)	−0.0025 (11)
C6	0.0150 (12)	0.0190 (14)	0.0250 (13)	0.0008 (10)	−0.0010 (10)	−0.0034 (11)
C4	0.0273 (14)	0.0247 (14)	0.0163 (12)	0.0099 (12)	−0.0010 (10)	−0.0035 (11)
C12	0.0143 (12)	0.0189 (13)	0.0177 (12)	−0.0005 (10)	0.0020 (9)	0.0003 (10)
C5	0.0175 (12)	0.0269 (15)	0.0221 (13)	0.0065 (11)	−0.0060 (10)	−0.0064 (11)

### Geometric parameters (Å, º)

**Table:**

Cu1—N2	2.014 (2)	C7—C12	1.398 (3)
Cu1—N4	2.020 (2)	C8—C9	1.389 (4)
Cu1—N1	2.022 (2)	C3—C4	1.386 (4)
Cu1—N3	2.023 (2)	C3—C2	1.398 (4)
Cu1—O1	2.2433 (18)	C3—H3	0.93
S2—O2	1.4783 (18)	C11—C12	1.391 (4)
S2—O4	1.4817 (18)	C11—C10	1.395 (4)
S2—O1	1.4818 (19)	C11—H11	0.93
S2—O3	1.4827 (18)	C9—C10	1.389 (4)
N3—C2	1.453 (3)	C9—H9	0.93
N3—H3A	0.9	C1—C2	1.386 (4)
N3—H3B	0.9	C1—C6	1.391 (4)
N4—C1	1.456 (3)	C10—H10	0.93
N4—H4A	0.9	C6—C5	1.386 (4)
N4—H4B	0.9	C6—H6	0.93
N1—C7	1.451 (3)	C4—C5	1.399 (4)
N1—H1A	0.9	C4—H4	0.93
N1—H1B	0.9	C12—H12	0.93
N2—C8	1.457 (3)	C5—H5	0.93
N2—H2A	0.9	O1WA—O1WC	0.777 (10)
N2—H2B	0.9	O1WA—O1WB	1.530 (11)
C7—C8	1.391 (3)	O1WB—O1WC	0.763 (11)
			
N2—Cu1—N4	177.02 (9)	Cu1—N2—H2B	109.6
N2—Cu1—N1	85.04 (8)	H2A—N2—H2B	108.2
N4—Cu1—N1	94.02 (8)	C8—C7—C12	120.4 (2)
N2—Cu1—N3	95.41 (8)	C8—C7—N1	117.6 (2)
N4—Cu1—N3	84.88 (8)	C12—C7—N1	122.0 (2)
N1—Cu1—N3	166.90 (9)	C9—C8—C7	120.0 (2)
N2—Cu1—O1	89.99 (8)	C9—C8—N2	122.9 (2)
N4—Cu1—O1	92.89 (8)	C7—C8—N2	117.2 (2)
N1—Cu1—O1	94.26 (8)	C4—C3—C2	119.9 (3)
N3—Cu1—O1	98.82 (8)	C4—C3—H3	120.1
O2—S2—O4	109.16 (10)	C2—C3—H3	120.1
O2—S2—O1	109.79 (11)	C12—C11—C10	120.1 (2)
O4—S2—O1	110.08 (11)	C12—C11—H11	119.9
O2—S2—O3	109.68 (11)	C10—C11—H11	119.9
O4—S2—O3	109.30 (11)	C8—C9—C10	119.9 (2)
O1—S2—O3	108.81 (10)	C8—C9—H9	120
S2—O1—Cu1	124.94 (10)	C10—C9—H9	120
C2—N3—Cu1	109.83 (15)	C2—C1—C6	120.6 (2)
C2—N3—H3A	109.7	C2—C1—N4	117.2 (2)
Cu1—N3—H3A	109.7	C6—C1—N4	122.2 (2)
C2—N3—H3B	109.7	C1—C2—C3	119.8 (2)
Cu1—N3—H3B	109.7	C1—C2—N3	117.7 (2)
H3A—N3—H3B	108.2	C3—C2—N3	122.5 (2)
C1—N4—Cu1	109.96 (15)	C9—C10—C11	120.2 (2)
C1—N4—H4A	109.7	C9—C10—H10	119.9
Cu1—N4—H4A	109.7	C11—C10—H10	119.9
C1—N4—H4B	109.7	C5—C6—C1	119.6 (3)
Cu1—N4—H4B	109.7	C5—C6—H6	120.2
H4A—N4—H4B	108.2	C1—C6—H6	120.2
C7—N1—Cu1	109.78 (15)	C3—C4—C5	119.9 (3)
C7—N1—H1A	109.7	C3—C4—H4	120
Cu1—N1—H1A	109.7	C5—C4—H4	120
C7—N1—H1B	109.7	C11—C12—C7	119.3 (2)
Cu1—N1—H1B	109.7	C11—C12—H12	120.3
H1A—N1—H1B	108.2	C7—C12—H12	120.3
C8—N2—Cu1	110.08 (15)	C6—C5—C4	120.2 (2)
C8—N2—H2A	109.6	C6—C5—H5	119.9
Cu1—N2—H2A	109.6	C4—C5—H5	119.9
C8—N2—H2B	109.6		
			
O2—S2—O1—Cu1	−48.22 (16)	N1—C7—C8—N2	1.2 (3)
O4—S2—O1—Cu1	71.97 (15)	Cu1—N2—C8—C9	−177.5 (2)
O3—S2—O1—Cu1	−168.27 (12)	Cu1—N2—C8—C7	3.4 (3)
N2—Cu1—O1—S2	128.22 (14)	C7—C8—C9—C10	−0.2 (4)
N4—Cu1—O1—S2	−51.05 (14)	N2—C8—C9—C10	−179.3 (2)
N1—Cu1—O1—S2	43.19 (14)	Cu1—N4—C1—C2	5.9 (3)
N3—Cu1—O1—S2	−136.31 (13)	Cu1—N4—C1—C6	−175.0 (2)
N2—Cu1—N3—C2	−172.48 (16)	C6—C1—C2—C3	−1.4 (4)
N4—Cu1—N3—C2	4.55 (16)	N4—C1—C2—C3	177.8 (2)
N1—Cu1—N3—C2	−81.2 (4)	C6—C1—C2—N3	178.6 (2)
O1—Cu1—N3—C2	96.69 (16)	N4—C1—C2—N3	−2.2 (3)
N1—Cu1—N4—C1	161.29 (16)	C4—C3—C2—C1	−0.2 (4)
N3—Cu1—N4—C1	−5.62 (16)	C4—C3—C2—N3	179.8 (2)
O1—Cu1—N4—C1	−104.23 (16)	Cu1—N3—C2—C1	−2.6 (3)
N2—Cu1—N1—C7	5.41 (16)	Cu1—N3—C2—C3	177.4 (2)
N4—Cu1—N1—C7	−171.75 (16)	C8—C9—C10—C11	0.4 (4)
N3—Cu1—N1—C7	−87.1 (4)	C12—C11—C10—C9	−0.4 (4)
O1—Cu1—N1—C7	95.04 (16)	C2—C1—C6—C5	1.9 (4)
N1—Cu1—N2—C8	−4.83 (16)	N4—C1—C6—C5	−177.2 (2)
N3—Cu1—N2—C8	162.02 (16)	C2—C3—C4—C5	1.2 (4)
O1—Cu1—N2—C8	−99.11 (16)	C10—C11—C12—C7	0.3 (4)
Cu1—N1—C7—C8	−5.1 (3)	C8—C7—C12—C11	−0.1 (4)
Cu1—N1—C7—C12	176.9 (2)	N1—C7—C12—C11	177.8 (2)
C12—C7—C8—C9	0.1 (4)	C1—C6—C5—C4	−0.9 (4)
N1—C7—C8—C9	−177.9 (2)	C3—C4—C5—C6	−0.7 (4)
C12—C7—C8—N2	179.2 (2)		

### Hydrogen-bond geometry (Å, º)

**Table:**

*D*—H···*A*	*D*—H	H···*A*	*D*···*A*	*D*—H···*A*
N1—H1*A*···O4^i^	0.90	2.03	2.904 (3)	164
N1—H1*B*···O2	0.90	2.06	2.873 (4)	149
N2—H2*A*···O3^ii^	0.90	2.16	3.059 (4)	174
N2—H2*B*···O3^iii^	0.90	2.02	2.890 (4)	161
N3—H3*A*···O3^iii^	0.90	2.45	3.188 (4)	139
N3—H3*A*···O4^iii^	0.90	2.24	3.068 (4)	153
N4—H4*A*···O4	0.90	2.37	3.202 (4)	153
N4—H4*B*···O2^i^	0.90	1.96	2.825 (4)	160
C4—H4···O4^iv^	0.93	2.59	3.512 (4)	172

Symmetry codes: (i) −*x*, *y*+1/2, −*z*+1/2; (ii) −*x*+1/2, *y*+1/2, *z*; (iii) *x*, *y*+1, *z*; (iv) −*x*, −*y*+1, −*z*.
